# Successful treatment of massive ascites due to lupus peritonitis with hydroxychloroquine in old- onset lupus erythematosus

**DOI:** 10.11604/pamj.2014.18.165.2080

**Published:** 2014-06-19

**Authors:** Sonia Hammami, Fethia Bdioui, Afef Ouaz, Hichem Loghmari, Sylvia Mahjoub, Hamouda Saffar

**Affiliations:** 1 Department of Internal Medicine, Fattouma Bourguiba Universitary Hospital, Monastir, Tunisia; 2 Department of Hepathology and Gastroenterology, Fattouma Bourguiba Universitary Hospital, Monastir, Tunisia

**Keywords:** Ascites, systemic lupus erythematosus, Hydroxychloroquine, Old-onset

## Abstract

Systemic lupus erythematous (SLE) is an auto-immune disease with multiple organ involvements that occurs mainly in young women. Literature data suggest that serositis is more frequent in late-onset SLE. However, peritoneal serositis with massive ascites is an extremely rare manifestation. We report a case of old-onset lupus peritonitis treated successfully by Hydroxychloroquine. A 77-year-old Tunisian woman was hospitalized because of massive painful ascites. Her family history did not include any autoimmune disease. She was explored 4 years prior to admission for exudative pleuritis of the right lung without any established diagnosis. Physical examination showed only massive ascites. Laboratory investigations showed leucopenia: 3100/mm^3^, lymphopenia: 840/mm^3^ and trace protein (0.03g/24h). Ascitic fluid contained 170 cells mm^3^ (67% lymphocytes), 46 g/L protein, but no malignant cells. The main etiologies of exudative ascites were excluded. She had markedly elevated anti-nuclear antibody (ANA) titer of 1/1600 and a significantly elevated titer of antibody to double-stranded DNA (83 IU/mL) with hypo-complementemia (C3 levl was at 67 mg/dL). Antibody against the Smith antigen was also positive. Relying on these findings, the patient was diagnosed with SLE and treated with Hydroxychloroquine 200 mg daily in combination with diuretics. One month later, there was no detectable ascitic fluid and no pleural effusions. Five months later she remained free from symptoms while continuing to take chloroquine. This case was characterized by old age of onset of SLE, the extremely rare initial presentation with lupus peritonitis and massive painful ascites with dramatic response to only hydroxychloroquine treatment.

## Introduction

Systemic lupus erythematous (SLE) is an auto-immune disease with multiple organ involvements that occurs mainly in young women. However, the elderly onset of SLE is rarely reported. This later age at onset has a strong modifying effect on the clinical presentation [1]. For instance, literature data suggest that pulmonary involvement and serositis are more frequent in late-onset SLE [2]. However, peritoneal serositis with massive ascites (known as lupus peritonitis) is an extremely rare manifestation [3]. Here, we report a 77-year-old woman with SLE whose disease manifested first as massive ascites treated successfully with Hydroxychloroquine.

## Patient and observation

A 77-year-old Tunisian woman was hospitalized because of massive painful ascites. Her family history did not include any autoimmune disease. She denied a history of hepatitis, jaundice or alcohol use. She had a history of diabetes, hypertension treated by glinide and calcium blocker. She was explored 4 years prior to admission for exudative pleuritis of the right lung without any established diagnosis after multiple explorations including thoracoscopy with biopsies. On admission, blood pressure was 150/70 mmHg, her rate was 80 /mn and body temperature was 37°C. The physical examination showed only abdominal distension related to the important ascites without collateral venous circulation. She had no skin lesions, lymphadenopathy, or hepatosplenomegaly and lower extremities showed no edema. Laboratory investigations showed: leucopenia with white blood cell count = 3100/mm^3^, lymphopenia = 840/mm^3^, hemoglobin = 10.5g/dL; low serum albumin level = 29g/L, 47;-globulin = 19g/L. blood glucose levels = 180 mg/dl and hemoglobin A1c = 8.6%. Urine analysis showed trace protein (0.03g/24h). Platelet count, activated partial thromboplastin time, liver function tests, blood urea nitrogen, serum creatinine, erythrocyte sediment rate and total cholesterol were within normal limits. Tests for HBsAg and HCV were negative. Abdominal ultrasonography showed ascitic fluid without any sign of bowel loop thickening or enteritis. Neither lymphadenopathy nor liver abnormality was present. A chest X-ray film disclosed bilateral pleural effusion. Ascitic obtained by aspiration contained 170 cells mm^3^ (67% lymphocytes), 46g/L protein, but no malignant cells. Cultures for bacteria and mycobacteria gave no growth. Tuberculosis investigation including initial tuberculin skin test and the research of Koch bacilli in sputum and urine were negative. Echocardiogram and electrocardiogram were normal. Computed tomographic scan showed massive ascites, no dysmorphic liver, and a mild bilateral pleural effusion. Pelvic examination was normal. Oesogastroduodenoscopy showed hiatal hernia without oesophageal varices. An exploratory laparoscopy was performed, revealing a large amount of ascite, a normal liver appearance, and no granulations suggesting tuberculosis or peritoneal carcinosis. Histological examination of the peritoneum showed non specific chronic inflammation. Systemic lupus erythematosus was suspected based on pleuritis, lymphopenia and leucopenia. An additional serologic survey revealed markedly elevated anti-nuclear antibody (ANA) titer of 1/1600 and a significantly elevated titer of antibody to double-stranded DNA (83 IU/mL; normal < 30 IU/mL). She had also positive serum antibody against the Smith antigen and low serum level of C3 complement component: 67 mg/dL (serum normal range: 84 – 151). Relying on these findings, the patient was diagnosed with SLE since 4 of the 11 diagnostic criteria of the American College of Rheumatology were met. The SLEDAI score according to Systemic Lupus Erythematosus Disease Activity Index was estimated to be 7 on admission. Hydroxychloroquine 200 mg daily in combination with diuretics was initiated. One month later, there was no detectable ascitic fluid and no pleural effusions. Five months later she remained free from symptoms while continuing to take Hydroxychloroquine

## Discussion

Systemic lupus erythematosus is an autoimmune disorder characterized by involvement of various organs. Inflammation of serous membranes including pericardium, pleura is relatively common (12%) and was admitted by American College of Rheumatology as one of the 11 criteria of SLE [4]. The incidences of pleuritis and pericarditis were reported to be higher in the elderly patients with SLE compared to younger patients [1]. Ascites may present with or without pain, and may be due to lupus peritonitis. In post mortem study, peritoneal involvement has been found in tow thirds of patients [5]. While, peritoneal serositis with ascites in clinical practice is extremely rare [6]. Diagnosing lupus peritonitis as initial symptoms of SLE remains a challenging task. Lupus peritonitis may have similar symptoms as those of acute abdominal conditions such as acute cholecystitis, acute pancreatitis, bleeding peptic ulcer, intestinal obstruction, peritonitis or rapid onset of massive ascites. It can also manifest in a chronic way as long lasting painless ascites, chronic pancreatitis or mild abdominal pain [7].

The mechanism of ascites in SLE may be multifactorial. First it's postulated that the deposition of immune-complexes in the peritoneum and activation of complements play in important role. Second, vasculitis of peritoneal vessels or the serous membrane of abdominal organs may be related to lupus peritonitis [5]. Corbella et al suggested that antiphospholipid emphasized chronic ascites in their patient [8].

Lupus ascites should be considered an exclusion diagnosis, identifying the nature of ascites remains a necessity as etiologies other than SLE should be ruled out, such as neoplasm: peritoneal carcinomatosis, primary masothelioma, hepatocell carcinoma; peritoneal infection like tuberculosis and HIV-associated peritonitis; nephritic syndrome, protein losing enteropathy, malnutrition and congestive heart failure [9]. Peritonel histology showed chronic inflammation and small vasculitis, the degree of inflammation in the peritoneum may be different between acute and chronic lupus [10].

In the case reported here, the patient had a massive painful ascites of acute–onset in a case of elderly onset lupus erythematosus. Her initial SLE manifestation may be pleuritis 4 years before. In this age, the presenting manifestations in these patients are commonly atypical and the diagnosis in this age group is usually tardy [1]. A literature search with Pubmed revealed only three elderly cases with lupus peritonitis were reported previously [8, 10, 11] Table 1. Proteinuria was seen in two patients, pleuretis was noted in all cases that presented chest X-ray including the present case. Pericarditis was fond in two cases. Steroid administration was performed in all patients. Two patients died despite treatment with high dose of prednisolone, Recently, Ito et al reported only three death from 16 cases reported, two from three death are elderly aged more than 70 years. They suggest that lupus peritonitis at elderly onset shows a poor prognosis than in the younger patients [10]. Some data showed a greater disease activity score and greater damage in late-onset patients, this hypothesis was supported by a higher mean of SLEDAI score and greater mortality [12]. More recently in updated pooled analysis data in the literature, Chen et al reported that old-onset SLE presented less skin and renal damages as well as autoantibodies, but lung damage was more severe [13]. The cause of death in elderly SLE patient may be multifactorial: Comorbidities concomitant therapies, infections, cardiovascular disorders and malignancies. In our case, the activity of SLE was low, SLEDAI was 7. The patient had a history of diabetes mellitus and her blood glucose still fluctuated. Considering her age and serious comorbidities, we initiate hydroxychloroquine, the massive ascites responded dramatically to this therapy. In old-onset SLE, the use of antimalarial agents such as hydroxychloroquine is an important aspect of SLE treatment when skin changes and arthritis are manifested, unless contraindicated. Willis et al confirmed in multiethnic multi-center cohort that Hydroxychloroquine therapy resulted in significant clinical improvement in SLE patients with reductions in IFN-a levels [14], Other treatments mostly include corticosteroids and immunosuppressive agents, depending on which organs are involved. Ito and associates in a recent review of chronic lupus peritonitis, reported that all patients treated by steroids, but only 13/16 reached remissions, In 40% additional immunosuppressant agents were associated to manage incomplete remission and recurrence ascites [10]. In our knowledge this the first case treated only by using hydroxychloroquine and diuretics. This suggests that inflammation of the peritoneum may be mild in the elderly.

**Table 1:** Literature Reporting of Lupus Peritonitis in the elderly (aged more than 60 years)

Author	case	Immunological findings	Proteinuria	Pleuritis	Pericarditis	Therapy	Outcome
**1.Corbella**	72/F	ANA, 1:640; ds-DNA, 29.3 U/ml;C3: 65.2 mg/dl; C4, normal	−	nd	nd	60 mg of PSL	Deceased
**2.Ito**	77/F	ANA, 2 + ; ds-DNAAb, 71 U/ml; C3, 20.4 mg/dl; C4: 9.4mg/dl	+	+	+	60 mg of PSL MPSL	Deceased
**3.Nakamura**	64/F	ANA, +; ds-DNA, +	+	+	+	MPSL + ECA	Remission
**4.Our case**	77/F	ANA, 1:1600; ds-DNA, 83 U/ml; C3, 67 mg/dl; C4: normal	−	+	−	HCQ	Remission

ANA:antinuclear antibody, ds-DNA: anti-DNA antibody, n.d.: no description. ECA: extracorporeal apheresis, HCQ: hydroxychloroquine

## Conclusion

This case we report is one of four cases at the onset age of SLE over 60 years, which presented with lupus peritonitis as initial symptom with a dramatic response with hydroxychloroquine. Systemic lupus erythematous rarely presents with massive ascites. Other causes of ascites should be excluded. Treatment is based on the use of diuretics, high dose of steroids, and in non-responding cases immunosuppressive drugs. Antimalarial agents might be effective in severe ascites if no acute life-threatening organ damage exists.
